# Human Extravillous Trophoblasts Require SRC-2 for Sustained Viability, Migration, and Invasion

**DOI:** 10.3390/cells14131024

**Published:** 2025-07-04

**Authors:** Vineet K. Maurya, Pooja Popli, Bryan C. Nikolai, David M. Lonard, Ramakrishna Kommagani, Bert W. O’Malley, John P. Lydon

**Affiliations:** 1 Department of Molecular and Cellular Biology, Baylor College of Medicine, Houston, TX 77030, USA; vineet.maurya@bcm.edu (V.K.M.); nikolai@bcm.edu (B.C.N.); dlonard@bcm.edu (D.M.L.); berto@bcm.edu (B.W.O.); 2 Department of Pathology and Immunology, Baylor College of Medicine, Houston, TX 77030, USA; pooja.popli@bcm.edu (P.P.); ramakrishna.kommagani@bcm.edu (R.K.)

**Keywords:** steroid receptor coactivator-2 (SRC-2), human extravillous trophoblast, HTR-8/SVneo, transcriptomics, WNT family member 9A (WNT 9A)

## Abstract

Defective placentation is a recognized etiology for several gestational complications that include early pregnancy loss, preeclampsia, and intrauterine growth restriction. Sustained viability, migration, and invasion are essential cellular properties for embryonic extravillous trophoblasts to execute their roles in placental development and function, while derailment of these cellular processes is linked to placental disorders. Although the cellular functions of extravillous trophoblasts are well recognized, our understanding of the pivotal molecular determinants of these functions is incomplete. Using the HTR-8/SVneo immortalized human extravillous trophoblast cell line, we report that steroid receptor coactivator-2 (SRC-2), a coregulator of transcription factor-mediated gene expression, is essential for extravillous trophoblast cell viability, motility, and invasion. Genome-scale transcriptomics identified an SRC-2-dependent transcriptome in HTR-8/SVneo cells that encodes a diverse spectrum of proteins involved in placental tissue development and function. Underscoring the utility of this transcriptomic dataset, we demonstrate that WNT family member 9A (WNT 9A) is not only regulated by SRC-2 but is also crucial for maintaining many of the above SRC-2-dependent cellular functions of human extravillous trophoblasts.

## 1. Introduction

A temporary fetomaternal organ, the human placenta is essential for intrauterine fetal development and protection [1,2]. Following the histiotrophic-to-hemotrophic switch that occurs toward the end of the first pregnancy trimester [3], the placenta is solely responsible for providing gas exchange, nourishment, and immunoprotection to the developing fetus. Apart from serving as a diffusion barrier, separating the maternal and fetal circulations, the placenta promotes adaptive changes in maternal physiology and metabolism, including hormone production and hemodynamic adaptations [4,5,6].

During early hemochorial placentation, the trophoblast cell lineage of the placenta expands and differentiates into villous and extravillous trophoblast cell types, each with specialized functions [6]. Through cell fusion, a primary function of villous trophoblasts is to provide an outer multinucleated epithelial cell layer of syncytiotrophoblasts, which cover floating chorionic villi and directly contact the maternal arterial blood [7,8,9]. Extravillous trophoblasts migrate from anchoring chorionic villi to invade the maternal decidual basalis to the proximal third of the uterine myometrium [10]. Apart from anchoring the embryonic-derived placenta to the maternal uterine compartment, extravillous trophoblasts transform into endovascular trophoblasts, which, together with local endometrial immune cells [11,12], remodel decidual spiral arterioles into large diameter uteroplacental arteries. With high-conductance and low-pressure, uteroplacental arteries serve to route maternal blood flow to the intervillous space. This placental remodeling of maternal vasodilation is commonly impaired in preeclampsia and related pregnancy disorders [9,13,14,15,16,17,18,19,20,21]. Although normal migratory and invasive properties of extravillous trophoblast cells are a prerequisite to preventing these placental disorders, the key signals that control these specialized cellular functions remain poorly understood.

Steroid receptor coactivator-2 (SRC-2), also known as nuclear receptor coactivator 2 (NCOA2), is a member of the p160/SRC family of transcriptional coregulators, which also includes SRC-1 and SRC-3 [22]. Interacting with either nuclear receptor or non-nuclear receptor transcription factors, individual members of the p160/SRC family exert distinct contributions to a broad spectrum of physiologies and pathophysiologies [22], including male and female reproductive processes [23,24]. We previously demonstrated that SRC-2 is essential for decidualization of stromal cells of the maternal endometrium [25,26,27,28], a crucial stage of the embryo implantation process. Although these studies provide important insights into our understanding of SRC-2 in the maternal endometrium during the earliest stages of pregnancy, the role of embryonic-derived SRC-2 during this implantation period is unclear. This knowledge gap is conspicuous in light of previous reports indicating that other members of the p160/SRC family serve important functions in a number of extraembryonic cell types, which are essential for the development and function of the placenta [29,30,31].

To address this issue, we report here that SRC-2 is expressed in multiple cell types of term placenta tissue from healthy women, and that SRC-2 expression in the commonly used HTR-8/SVneo human trophoblast cell line is essential for trophoblastic cell viability, migration, and invasion. In addition to significant changes in expression of previously reported genes in extravillous trophoblasts and/or the HTR-8/SVneo cell line, genome-wide RNA sequencing (RNA-seq) revealed that the expression levels of WNT-related factors are markedly reduced in HTR-8/SVneo cells with decreased expression levels of SRC-2. Importantly, we identified WNT family member 9A (WNT 9A) as a critical SRC-2-responsive molecular target that is required for optimum execution of many of these key cellular processes, which are known to occur during hemochorial placentation.

## 2. Materials and Methods

### 2.1. Human Placenta Tissue

Placental specimens were obtained from Dr. Ramkumar Menon at the University of Texas Medical Branch (UTMB) in Galveston, Texas. Dr. Menon’s human protocol was prospectively approved by the Institutional Review Board (IRB, approval number IRB16.0058, acceptance date: 7 January 2020) at UTMB. Patient consent was exempted as placentae were non-identifiable and were to be discarded. Placentae were obtained from not-in-labor repeat elective Cesareans. For immunohistochemistry, placental samples were washed in phosphate-buffered saline (PBS) to remove blood clots before fixation in 10% neutral buffered formalin (NBF) for 24 h at 4 °C. Following graded alcohol washes, tissues were embedded in paraffin blocks, which in turn were sectioned to a thickness of 5 μm prior to placement onto microscope glass slides for immunohistochemical and dual immunofluorescence staining. For immunohistochemical staining for SRC-2, SRC-3, and WNT9A, the following primary antibodies were used, respectively: a rabbit polyclonal anti-human SRC-2 (#A300-345A; 1:500 dilution, Bethyl Laboratories, Montgomery, TX, USA); a rabbit monoclonal anti-human SRC-3 (#2126; 1:200 dilution Cell Signaling Technologies, Inc. Danvers, MA, USA), and a rabbit polyclonal anti-human WNT9A (#AB176973 1:100 dilution Abcam Inc., Waltham, MA, USA). For dual immunofluorescence staining for SRC-2 and the beta subunit of human chorionic gonadotropin (hCG), the following primary antibodies were used, respectively: a rabbit anti-SRC-2 primary antibody ((#96687s) Cell Signaling Technology Inc.) and a mouse anti-hCG beta-subunit primary antibody ((#ab9582) Abcam Inc.). For dual immunofluorescence staining, the following secondary antibodies were used: donkey anti-rabbit IgG conjugated with Alexa Fluor PLUS 594 (#A21207) and donkey anti-mouse IgG conjugated with Alexa Fluor PLUS 488 (#A21202), both purchased from ThermoFisher Scientific Inc., Waltham, MA, USA. Following staining, slides were coverslipped with Vectashield mounting medium containing 4′,6-diamidino-2-phenylindole (DAPI; (H-1200) Vector Laboratories, Inc., Burlingame, CA, USA).

### 2.2. Immortalized Human Extravillous Trophoblast Cell Line

Previously described [32,33,34,35,36], the HTR-8/SVneo immortalized human trophoblast cell line was purchased from the American Type Culture Collection (ATCC (#CRL-3271)), Manassas, VA, USA. Briefly, the HTR-8/SVneo cell line was originally derived from first-trimester human trophoblasts and immortalized by transfection with a gene encoding the Simian virus 40 large T antigen [32]. For studies described here, the HTR-8/SVneo cell line was cultured in phenol red-free RPMI-1640 medium, supplemented with 5% fetal bovine serum (FBS; Sigma-Aldrich, St. Louis, MO, USA) and a 1% penicillin–streptomycin antibiotic solution (ThermoFisher Scientific Inc., Waltham, MA, USA); medium was changed every other day.

### 2.3. Transfection of Small Interfering RNAs

For siRNA-mediated knockdown of gene expression, HTR-8/SVneo cells were cultured in six-well plates before transfection with 60 picomoles of one of the following siRNAs: non-targeting (NT (control)) siRNA ((D-001810-10-05) Dharmacon Inc., Lafayette, CO, USA), siRNAs targeting SRC-2 ((L-020159-00-0005) Dharmacon Inc.) or Wnt 9A ((L-008392-00-0005) Dharmacon Inc.). Under serum-starved conditions, transfections were performed using the Lipofectamine RNAi MAX transfection reagent (Invitrogen Corporation, Carlsbad, CA, USA). Forty-eight hours post-transfection, cells were harvested for quantitative real-time PCR (qRT-PCR), RNA-sequencing (RNA-seq), or Western analysis. Alternatively, transfected HTR-8/SVneo cells were trypsinized and re-plated for assays of cell viability, clonogenic survival, migration, or invasion competency.

### 2.4. Quantitative Real-Time PCR

Cells were lysed in RNA lysis buffer before total RNA was isolated with the RNAeasy Plus Mini Kit (#74134, Qiagen Inc., Germantown, MD, USA) or the Purelink RNA Mini Kit (#12183020, ThermoFisher Scientific Inc.). A NanoDrop 2000 UV/Vis spectrophotometer (ThermoFisher Scientific Inc.) was used for RNA quantification. Total RNA (1 µg) was reverse transcribed using the High-Capacity cDNA Reverse Transcription Kit (#4368814, ThermoFisher Scientific Inc.). Synthesized cDNA was diluted to 10 ng/µL before qRT-PCR was performed using Fast TaqMan 2× Mastermix (Applied Biosystems/Life Technologies, Grand Island, NY, USA); TaqMan assays used in this study are listed in Table 1. All qRT-PCR experiments were performed using the 7500 Fast Real-time PCR system (Applied Biosystems/Life Technologies, Grand Island, NY, USA). For quantitation of gene expression changes, five replicate samples per treatment group (i.e., *NT* siRNA or *SRC-2* siRNA) were used. To normalize for sample-to-sample variation, the ΔCt value was calculated using the formula: Ct (gene-of-interest)-Ct (*Hypoxanthine guanine phosphoribosyl transferase* (*HPRT*), a housekeeping gene). For each replicate, the ΔΔCt value for a gene-of-interest was calculated using the formula: ΔCt (the experimental sample (i.e., *NT* siRNA or *SRC-2* siRNA))-ΔCt (the corresponding reference sample (i.e., *NT* siRNA)). Per replicate, the gene expression fold change (FC) was calculated from the ΔΔCt value using the formula 2^Λ−ΔΔCt^. The average gene expression FC for a gene-of-interest from the five replicates per treatment was the value plotted on the Y-axis of the resultant histograms. Values from the 2^Λ−ΔΔCt^ formula were log transformed in Excel to enable follow-up statistical analysis.

### 2.5. Genome-Scale RNA Expression Profiling

Genome-wide RNA-seq analysis was performed as previously described [37,38]. Briefly, total RNA purity and integrity were measured using a NanoDrop spectrophotometer (ThermoFisher Scientific Inc.) and a 2100 Bioanalyzer with RNA chips (Agilent Technologies, Santa Clara, CA, USA), respectively. Only RNA samples with an RNA integrity score greater than 9 were included; three replicate RNA samples were used for each experimental group. Sequencing libraries were prepared from 250 ng of RNA using the TruSeq Stranded mRNA kit (Illumina Inc., San Diego, CA, USA) and amplified by PCR. Quality analysis of resultant libraries was performed on a 4200 TapeStation with D1000 ScreenTape assays (Illumina Inc.). The adapter-ligated fragment concentration was estimated by qRT-PCR with a KAPA Library Quantification Kit (KAPA Biosystems, Wilmington, MA, USA). After equimolar pooling, libraries were quantified using a 2100 Bioanalyzer (with a High Sensitivity DNA Kit and DNA chips); libraries were sequenced on the NovaSeq 6000 platform (Illumina Inc.). Paired-end 100 base-pair sequencing reads were generated at mid-output, and filtered reads (in FASTQ file format) were aligned to the human genome (Genome Reference Consortium Human Build hg38; National Center for Biotechnology Information (NCBI)) using the universal RNA-seq aligner HISAT2 [39]. The Python-based software package HTSeq was used to determine the number of reads that aligned to known genes (http://www-huber.embl.de/users/anders/HTSeq, accessed on 7 May 2024) [40]. To reduce possible PCR bias, read duplicates were removed with Picard Tools (https://broadinstitute.github.io/picard, accessed on 7 May 2024).

To identify genes that were differentially expressed between the two treatment groups, the R package DESeq was applied to the gene expression data [41]. The false discovery rate (FDR) of differentially expressed genes (DEGs) was estimated using the Benjamini and Hochberg method [42]. Gene expression comparisons with an FDR ≤ 0.05 and an absolute fold change (IFCI) ≥ 1.3 were considered significant [43]. Fragments per kilobase of transcript per million (FPKM) values of transcripts were used for hierarchical clustering. Clustered heatmaps were generated with the pheatmap package in R. With raw gene count data, principal component analysis (PCA) was performed with the R function prcomp package (https://cran.r-project.org). All raw data files were deposited in the Gene Expression Omnibus (GEO) repository at NCBI (www.ncbi.nlm.nih.gov/geo (GSE: 278677)). Gene ontology enrichment analysis was performed using the Database for Annotation, Visualization, and Integrated Discovery (DAVID) functional annotation clustering tool (https://david.ncifcrf.gov/) [44]. Biological pathways involving genes that are overrepresented in datasets of DEGs were identified using the Kyoto Encyclopedia of Genes and Genomes (KEGG; https://www.genome.jp/kegg/) [45]. For KEGG pathway enrichment analysis, the KEGG Orthology-Based Annotation System (KOBAS 2.0) was used [46]; CD Genomics (Shirley, New York, NY, USA) performed the majority of the bioinformatics analysis.

### 2.6. Immunoblotting

Proteins (20 μg) from cell lysates were resolved on 4–15% and 10% sodium dodecyl sulfate polyacrylamide gels and transferred to polyvinylidene difluoride (PVDF) membranes. Following protein transfer, the PVDF membranes were blocked for 1 h with 5% non-fat dry milk (sc-2324 (Blotto), Santa Cruz Biotechnology Inc., Dallas, TX, USA) in Tris-buffered saline with Tween 20 (TBS-T) and incubated overnight at 4 °C with the following primary antibodies diluted in 5% non-fat milk in TBS-T: anti-SRC-2 (1:2000; #610984, BD Transduction Laboratories, Inc., East Rutherford, NJ, USA), anti-SRC-1 (1:1000; #2191 Cell Signaling Technology, Inc., Danvers, MA, USA); anti-SRC-3 (1:1000, #2126 Cell Signaling Technology Inc.); anti-HLA-G (1:1000; #NBP3-20480, Novus Biologicals LLC, Centennial, CO, USA; anti-WNT 9A (1:1000; #ab176973, Abcam, Eugene, OR, USA), and anti-β-actin (1:10,000; ##4970, Cell Signaling Technology, Inc.). The blots were then probed for 1 h at room temperature with horseradish peroxidase (HRP)-conjugated anti-rabbit (1:5000; A27036, ThermoFisher Scientific Inc.) or anti-mouse IgG secondary antibodies (1:10,000; #7076, Cell Signaling Technology, Inc.) diluted in 5% non-fat milk in TBS-T. Chemiluminescence was detected with the SuperSignal West Pico PLUS Chemiluminescent Substrate (#1863097, ThermoFisher Scientific, Inc.) and digitally imaged using the Bio-Rad ChemiDoc imaging system (Bio-Rad Laboratories, Hercules, CA, USA).

### 2.7. Cell Viability Assay

Cells were seeded in triplicate wells in 96-well culture plates at a density of 5 × 10^3^ cells per well. Cells transfected with siRNAs for 48 h were further cultured for 0, 24, 48, 72, or 96 h before the number of viable cells was assessed using the CellTiter 96^®^ Non-Radioactive Cell Proliferation Assay kit (#G4000, Promega Inc., Madison, WI, USA). Briefly, after a specific time in culture, 15 mL of 3-(4, 5-dimethylthiazol-2-yl)-2, 5-diphenyltetrazolium bromide (MTT; Promega, Madison, WI, USA) was added to each well for a final concentration of 0.5 mg/mL. Cells were then incubated at 37 °C for an additional three hours in the dark. After the three-hour incubation period, the supernatant was removed, and the stop/solubilizing solution (dimethyl sulfoxide (100 µL)/well) was added to dissolve the formazan crystals. Cells were then incubated further for 15 min at 37 °C with gentle agitation. The absorbance of the final mixture was recorded at 570 nm (formazan absorbance maximum) using a 96-well microplate ELISA reader. Relative number of viable cells was calculated using the mean absorbance at the “N” time point divided by the mean absorbance at 0 h, where N = 24, 48, 72, or 96 h. Each experiment was repeated three times with three to five technical replicates for each treatment group.

### 2.8. Clonogenic Survival Assay

Forty-eight hours after siRNA transfection, cells were cultured in triplicate wells in six-well culture plates for 10 days (3 × 10^3^ cells per well), with the medium replaced every other day. After the 10-day culture period, cells were fixed with 4% paraformaldehyde for 15 min and washed with PBS for 10 min before colonies were stained with crystal violet solution (0.5%) for 15 min [26]. Stained colonies were photographed following de-staining in tap water and air-drying. The bound crystal violet was eluted by the addition of 30% acetic acid (300 μL) to each insert, followed by shaking for 10 min. The eluent from the wells was transferred to a 96-well clear microplate, and the absorbance at 590 nm was measured using a plate reader (Tecan Infinite M200 PRO, AG, Zurich, Switzerland). Each experiment was repeated three times with triplicate wells for each treatment group.

### 2.9. Transwell Cell Migration Assay

Cell migration was assessed using Corning transwell chambers (#3422, Corning Inc., Glendale, AZ, USA). Briefly, cells were seeded in six-well culture plates and cultured until reaching 70–80% confluency before siRNA transfection. Forty-eight hours after transfection under serum-starved conditions, the cells were trypsinized and resuspended in culture medium without fetal bovine serum (FBS). Cells were counted and then diluted at a density of 1 × 10^5^ cells/250 μL in Opti-MEM medium. Subsequently, the cell suspension was seeded into the upper chamber of each transwell. In the lower chamber, 600 μL culture medium with 10% FBS was used as a chemoattractant. The cells were cultured in a humidified incubator at 37 °C with 5% CO_2_ for 24 h. After 24 h, the transwell inserts were washed twice with phosphate-buffered saline (PBS); cells were fixed with 4% paraformaldehyde in PBS for 15 min. Cells on the inside of transwell inserts were gently removed using moistened cotton swabs, while cells retained on the lower surface of the membrane were stained with crystal violet for 10 min. The transwell inserts were washed twice with PBS to remove unbound crystal violet and then air-dried. The migrated cells were observed and imaged with an inverted phase-contrast microscope (EVOS^TM^ XL Core Imaging System, #AMEX1000, ThermoFisher Scientific Inc.). The bound crystal violet was eluted by adding 30% acetic acid (300 μL) onto each insert with shaking for 10 min. The eluent from the lower chamber was transferred to a 96-well clear microplate, and the absorbance at 590 nm was measured using a plate reader (Tecan Infinite M200 PRO). For each treatment group, experiments were performed three times with triplicate samples.

### 2.10. Transwell Cell Invasion Assay

The Corning BioCoat Matrigel Invasion Chamber Kit ((#354480) Corning Inc.) was used to quantitate cell invasion. Following siRNA transfection for 48 h under serum-starved conditions, cells were trypsinized and resuspended in culture medium without FBS. Cells were counted and then diluted at a density of 1 × 10^5^ cells/250 μL in Opti-MEM medium. Serving as a chemoattractant, culture medium with 10% FBS was added (600 μL) to the bottom of each transwell of the invasion chamber plate. Suspended cells (1 × 10^5^ cells/250 µL) were then added to each transwell insert and allowed to migrate. After 24 h, cells were removed from the top surface of the transwell using a cotton swab. Migrated cells were fixed with 4% paraformaldehyde in PBS for 15 min and stained with crystal violet solution for 10 min [37]. To remove unbound crystal violet stain, the transwell inserts were washed twice with PBS, air-dried, and then digitally imaged using a Zeiss stereomicroscope with an attached AxioCam MRC-5 digital camera (Zeiss, Jena, Germany). Bound crystal violet was eluted by adding 30% acetic acid (300 μL) into each insert with shaking on an orbital shaker for 10 min. The eluent from the lower chamber was transferred to a 96-well clear microplate; the absorbance at 590 nm was measured using a plate reader (Tecan Infinite M200 PRO). Each experiment was repeated three times with triplicate samples for each treatment group.

### 2.11. Statistical Analysis

To estimate the statistical significance of differences between the two treatment groups, two-tailed unpaired Student *t*-tests were used. Unless otherwise stated, data were graphically presented as the mean ± standard deviation. Differences with a *p*-value < 0.05 were considered statistically significant; asterisks represent the level of significance: * *p* < 0.05, ** *p* < 0.01, *** *p* < 0.001, and **** *p* < 0.0001. Prism software version 9 (GraphPad Software Inc., San Diego, CA, USA) was used for many of the reported statistical analyses.

## 3. Results

### 3.1. Human Term-Placenta Expresses SRC-2

**Figure 1:**
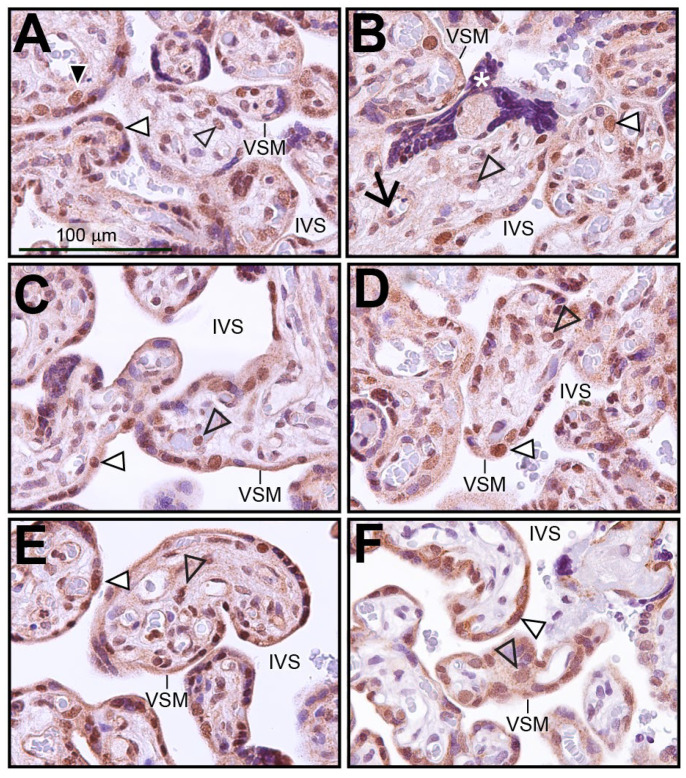
Expression of SRC-2 in human term-placental tissue: (**A**–**E**) Immunohistochemical staining shows the location of SRC-2 protein expression in chorionic villi of human term (39 weeks)-placental tissue from five subjects. The intervillous space (IVS) and the vasculosyntial membrane (VSM) demarcating maternal blood sinusoids are indicated. White triangle indicates a nucleus of the syncytiotrophoblast cell layer that is positive for SRC-2 expression. White asterisk highlights a syncytial knot, which is a prominent feature in the term placenta [47]. Expression of SRC-2 is also evident in sparsely distributed cytotrophoblasts ((**A**) (black triangle)), in perivascular fibroblasts of fetal blood vessels ((**B**) black arrow), stromal cells of the chorionic villus (open arrowhead), and in syncytiotrophoblasts (white triangle). (**F**) Immunohistochemical staining reveals a similar expression pattern for SRC-3. Scale bar in (**A**) applies to all panels.

Immunohistochemistry was performed on human term-placental tissue to identify the cell types within the chorionic villus that express SRC-2 (Figure 1A–E). Immunopositivity for SRC-2 was detected in the majority of syncytiotrophoblasts, which form the outer cell layer of the chorionic villus and are in direct contact with the maternal arterial blood in the intervillous space (IVS). This expression pattern was confirmed by dual immunofluorescence staining for SRC-2 and the syncytiotrophoblast marker, hCG (Figure S1). A smaller population of inner cytotrophoblast cells also exhibit SRC-2 immunopositivity (Figure 1A). Expression of SRC-2 was also observed in stromal cells within chorionic villi as well as in perivascular fibroblasts juxtaposed to fetal blood vessels (Figure 1A–E). Immunohistochemical staining for SRC-3 in human term-placental tissue shows a similar spatial expression pattern in the chorionic villi as SRC-2 (compare Figure 1A–E with Figure 1F).

### 3.2. The HTR-8/SVneo Cell Line Expresses SRC-2

**Figure 2:**
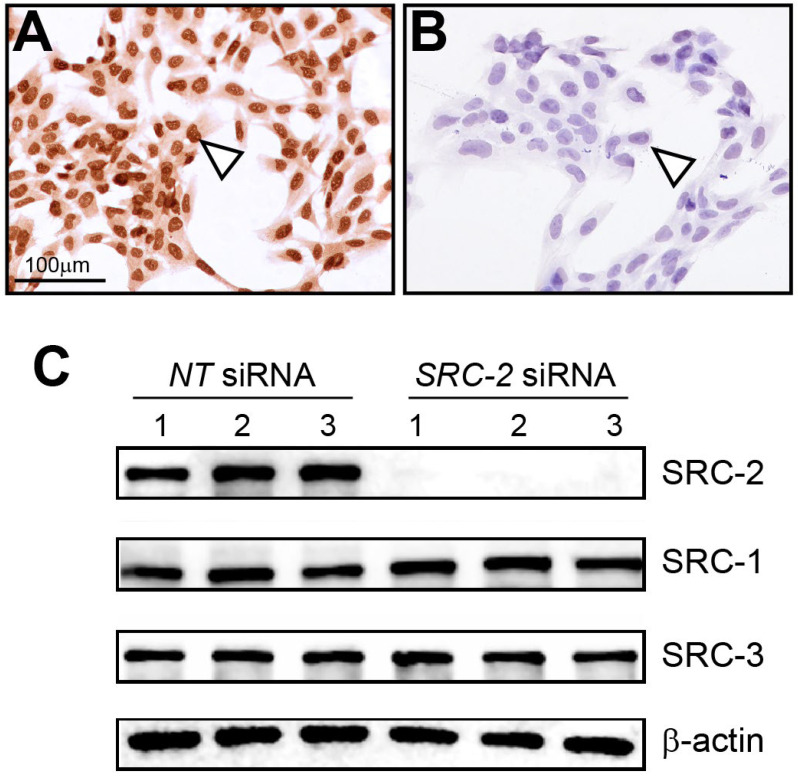
Expression of SRC-2 in HTR-8/SVneo cells: (**A**) Immunocytochemistry shows strong nuclear staining for SRC-2 expression in HTR-8/SVneo cells (white triangle). (**B**) Knockdown of SRC-2 results in negligible SRC-2 expression in HTR-8/SVneo cells (white triangle), demonstrating the specificity and efficacy of this siRNA-mediated gene knockdown approach. (**C**) Western immunoblot confirms both strong SRC-2 expression in HTR-8/SVneo cells (*NT* (control) siRNA group) and negligible SRC-2 expression (*SRC-2* siRNA group); lanes 1–3 represent replicates for HTR-8/SVneo cells transfected with either *NT* siRNA or siRNA targeting *SRC-2*. The expression levels of SRC-1 and SRC-3 are not altered in HTR-8/SVneo cells following *SRC-2* knockdown; β-actin is a loading control. Scale bar in panel (**A**) applies to panel (**B**).

Immunocytochemistry detected strong nuclear staining for SRC-2 expression in HTR-8/SVneo cells (Figure 2A). As validation for the above cell expression data, immunocytochemistry detected negligible SRC-2 expression in HTR-8/SVneo cells following siRNA-mediated knockdown of SRC-2 (Figure 2B), which was further confirmed at the molecular level by immunoblot analysis (Figure 2C). Importantly, the expression levels of SRC-1 and SRC-3 are not affected by SRC-2 knockdown. Together, these results show that SRC-2 is expressed in HTR-8/SVneo cells, which model extravillous trophoblasts, an important extra-embryonic cell type in early placenta development.

### 3.3. Sustained Viability, Motility, and Invasiveness of HTR-8/SVneo Cells Require Normal SRC-2 Expression Levels

**Figure 3:**
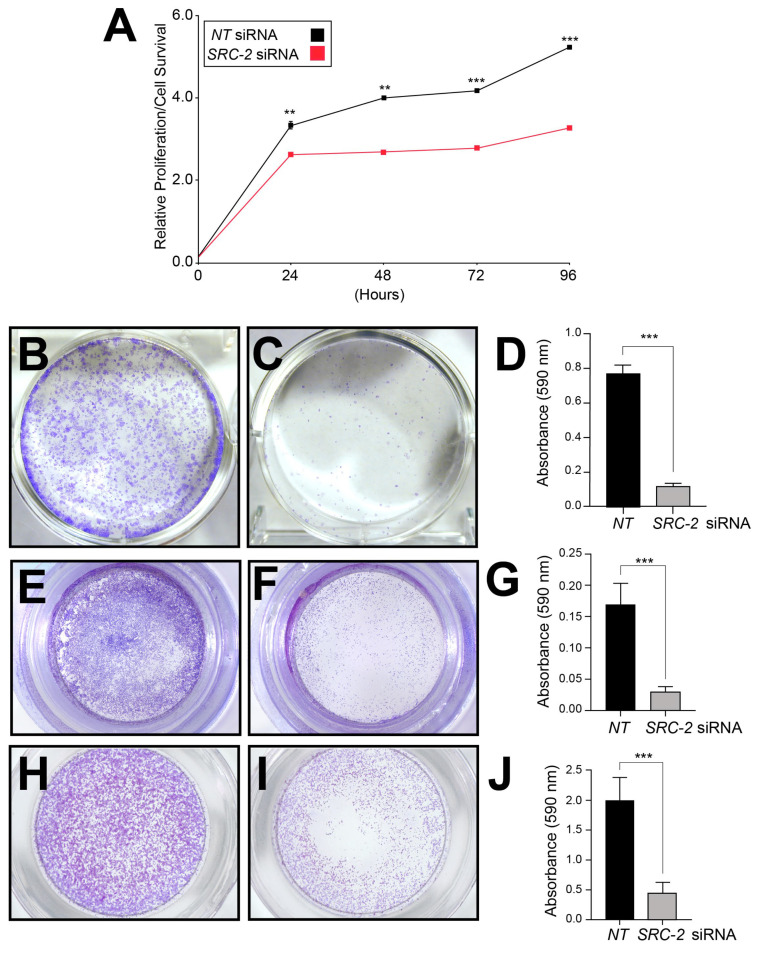
Sustained viability/proliferation, invasion, and migration of HTR-8/SVneo cells require SRC-2: (**A**) The MTT assay shows HTR-8/SVneo cells with an SRC-2 deficit (*SRC-2* siRNA transfected group) score for a significantly lower number of viable cells at each predetermined time point (24, 48, 72, and 96 h) in culture. (**B**) Crystal violet staining reveals numerous large colonies from HTR-8/SVneo cells previously transfected with *NT* siRNAs. The image is a representative result after the *NT* or *SRC-2* siRNA transfection period of 48 h and a post-transfection period of 12 h in culture. (**C**) Crystal violet staining shows a significant decrease in the number of colonies; colonies are also markedly smaller in size. (**D**) Histogram quantitatively displays the differences between the treatment groups in panels (**B**,**C**) based on the crystal violet dye absorbance reading at 590 nm. (**E**) Transwell migration assay shows the migration of numerous HTR-8/SVneo cells following *NT* siRNA transfection. (**F**) Transwell migration assay reveals a significant attenuation of the migratory ability of HTR-8/SVneo cells following *SRC-2* siRNA transfection. (**G**) Histogram represents the quantitation of the migratory differences between treatment groups shown in (**E**,**F**). (**H**) Transwell invasion assay shows significant invasive capability of HTR-8/SVneo cells following *NT* siRNA transfection. (**I**) Transwell invasion assay shows the striking reduction in invasive capability of the HTR-8/SVneo cell line following *SRC-2* siRNA transfection. (**J**) Histogram displays the quantitative differences between the treatment groups shown in panels (**H**,**I**). Results are indicated as the mean ± standard deviation and are representative of three independent experiments; ** *p*-value < 0.01; and *** *p*-value < 0.001.

Because extravillous trophoblasts rely on sustained viability as well as motile and invasive functions to establish and expand the utero-placental interface [1,2], and because SRC-2 is essential for these cellular functions in other cell types [48,49,50,51], we used a siRNA-mediated knockdown approach in conjunction with established viability/proliferation, clonogenic survival, and migration and invasion assays to determine whether SRC-2 is required for any or all of these cellular properties in HTR-8/SVneo cells (Figure 3).

The standard MTT-based cell viability/proliferation assay, a colorimetric assay that measures viable cell number, demonstrated that the number of viable HTR-8/SVneo cells is markedly reduced at each pre-determined time point in culture when *SRC-2* levels are reduced (Figure 3A). In clonogenic survival assays, the number and size of colonies arising from single HTR-8/SVneo cells were also significantly decreased following *SRC-2* knockdown (Figure 3B–D). Furthermore, transwell assays for cell migration and invasion revealed that SRC-2 is essential for both cellular processes in HTR-8/SVneo cells (Figure 3E–J). These results underscore the importance of SRC-2 in several critical cellular functions that are known to be required by extravillous trophoblasts during early development of the placenta [2].

### 3.4. Genome-Wide Changes in the HTR-8/SVneo Cell Transcriptome Following SRC-2 Depletion

Since SRC-2 is a transcriptional coregulator that controls transcriptome integrity in other cell types and tissues [25,27,38], coupled with the finding that SRC-2 is essential for a number of extravillous trophoblastic cellular functions required for placentation (Figure 2), we used RNA-seq analysis to identify the trophoblastic transcriptome in HTR-8/SVneo cells that is dependent on SRC-2 (Figure 3).

**Figure 4:**
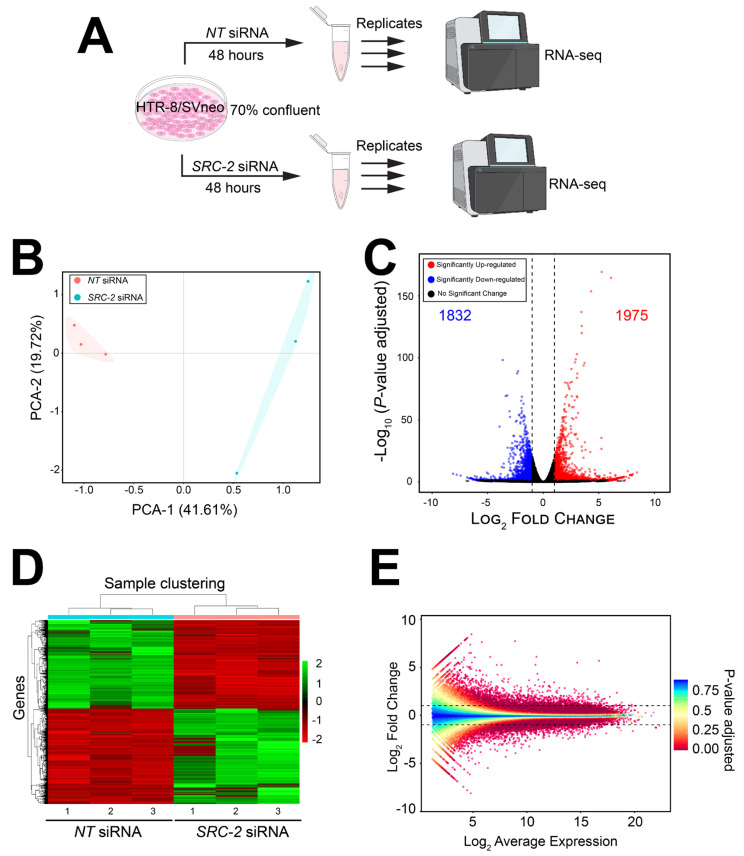
The HTR-8/SVneo cellular transcriptome is significantly changed following SRC-2 knockdown: (**A**) Schematic showing the key stages of the RNA-seq workflow; triplicate samples for the NT siRNA and SRC-2 siRNA treatment groups were used. (**B**) Principal component analysis (PCA) shows a clear separation between the triplicate NT siRNA and SRC-2 siRNA groups. (**C**) The volcano plot graphically displays the total number of genes differentially expressed between the NT siRNA and SRC-2 siRNA treatment groups; 1832 downregulated and 1975 upregulated genes are shown in blue and red, respectively. Significance (*p*-value) versus log_2_ fold change is plotted on the y- and x-axis, respectively. (**D**) The heatmap represents an unsupervised hierarchical clustergram of the total number of genes differentially expressed between the NT siRNA and SRC-2 siRNA groups that reached the FDR and IFCI thresholds of ≤0.05 and ≥1.3, respectively. Each row represents a gene, while each column denotes a sample replicate in each treatment group; the dendrogram on both axes shows the arrangement of the clusters following analysis. The intensity of the color indicates the level of expression for each gene in the sample replicate. The intensity in terms of the normalized expression values is indicated by the vertical color key on the right. (**E**) The MA plot shows the relationship between the log2 fold change and the mean normalized counts of DEGs in HTR-8/SVneo cells treated with NT siRNA or SRC-2 siRNA.

The experimental design for the RNA-seq study is schematically shown in Figure 4A. Briefly, HTR-8/SVneo cells cultured to 70% confluency in triplicate wells of six-well plates were transfected with *NT* or *SRC-2* siRNAs forty-eight hours prior to RNA isolation and sequencing (Figure 4A). Differentially expressed genes between the *NT* siRNA and *SRC-2* siRNA-transfected groups are listed in Table S1, which also includes the results of the gene ontology (GO) analyses. As expected, the expression of SRC-2 was significantly downregulated (log2 fold change: −1.95) in the HTR-8/SVneo cell group that was transfected with SRC-2 siRNAs (yellow highlighted row).

The FPKM values for all genes were analyzed by principal component analysis (PCA; (Figure 4B)). The PCA demonstrates a significant separation between the cell triplicates that were transfected with either *NT* siRNAs or *SRC-2* siRNAs (Figure 4B). Both a volcano plot and an expression heatmap provide a genome-wide perspective of the transcriptional changes that follow *SRC-2* knockdown (Figure 4C and Figure 3D, respectively). Attaining the predetermined FDR (≤0.05) and IFCI (≥1.3) cutoffs, the RNA-seq analysis identified 3807 DEGs (1832 upregulated and 1975 downregulated) in HTR-8/SVneo cells following *SRC-2* knockdown. The relationship between the log2 fold change and the mean normalized count of the DEG dataset is also graphically displayed in the MA plot (Figure 4E).

Initial gene ontology analysis revealed an enrichment for genes in the DEG dataset that is associated with general biological pathways involved in wound healing, extracellular signal-activated kinase 1 (ERK1) and ERK2 signaling, cytokine activity, and G-protein coupled receptor activity (Figure S2). In addition, KEGG pathway analysis of the DEGs showed an overrepresentation of pathways involved in phosphoinositide 3-kinase (PI3K)/AKT signaling, cell adhesion, and extracellular matrix (ECM)–receptor interactions (Figure S2). Interestingly, many of these enriched pathways are known to govern trophoblast cell physiology [52,53,54,55]. Together, these findings indicate that SRC-2 regulates biological responses that are in line with cellular phenotypes that underpin trophoblast functions.

### 3.5. Gene Expression Associated with Trophoblastic Biology and WNT Signaling Are Significantly Reduced in HTR-8/SVneo Cells Following SRC-2 Depletion

**Figure 5:**
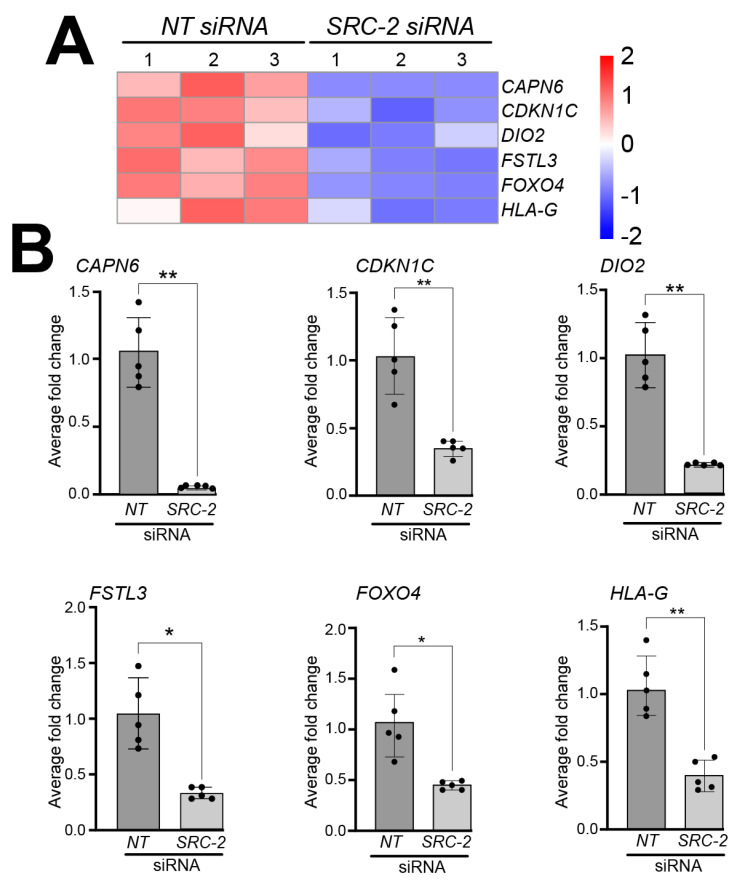
Knockdown of *SRC-2* in the HTR-8/SVneo cell line attenuates the expression levels of genes previously reported to be expressed in extravillous trophoblast cells: (**A**) The heat map displays the alterations in gene expression between *NT* siRNA and *SRC-2* siRNA replicates. The expression levels of calpain 6 (*CAPN6*); cyclin-dependent kinase inhibitor 1C (*CDKN1C*); iodothyronine deiodinase 2 (*DIO2*); follistatin-like 3 (*FSTL3*); forkhead box O4 (*FOXO4*); and human leukocyte antigen G (*HLA-G*) are significantly reduced in the *SRC-2* siRNA-treated group compared with the *NT* siRNA group. (**B**) Quantitative real-time PCR confirms the above-predicted trophoblast cell marker gene expression changes between the *NT* siRNA and *SRC-2* siRNA-treated groups. Results are indicated as the average fold change (±standard deviation) in expression of the gene of interest for each experimental condition (*NT* or *SRC-2* siRNA knockdown). The average fold change (FC) calculation is detailed in the Section 2. All results presented are representative of three independent experiments; * *p*-value < 0.05; ** *p*-value < 0.01.

Analyses of the HTR-8/SVneo cell DEG dataset also showed a significant reduction in the expression levels of the following genes that have previously been shown to be expressed in extravillous trophoblasts and/or HTR-8/SVneo cells; these include Calpain 6 (CAPN6) [56], Cyclin-dependent kinase inhibitor 1C (CDKN1C) [57], deiodinase type 2 (DIO2) [58], follistatin-like 3 (FSTL3) [59,60,61], forkhead box 4 (FOXO4) [62,63], and human leukocyte antigen-G (HLA-G) [64,65,66,67] (Figure 5A); expression validation of these genes by qRT-PCR is displayed in Figure 5B.

In addition, the dysregulated expression of the following trophoblast stem cell markers: Aldehyde dehydrogenase 1 family member A1 (ALDH1A1) [68], C-C motif chemokine receptor 7 (CCR7) [69], and laminin subunit beta-3 (LAMB3) [70] in HTR-8/SVneo cells following *SRC-2* knockdown is shown in Figure S3A,B.

**Figure 6:**
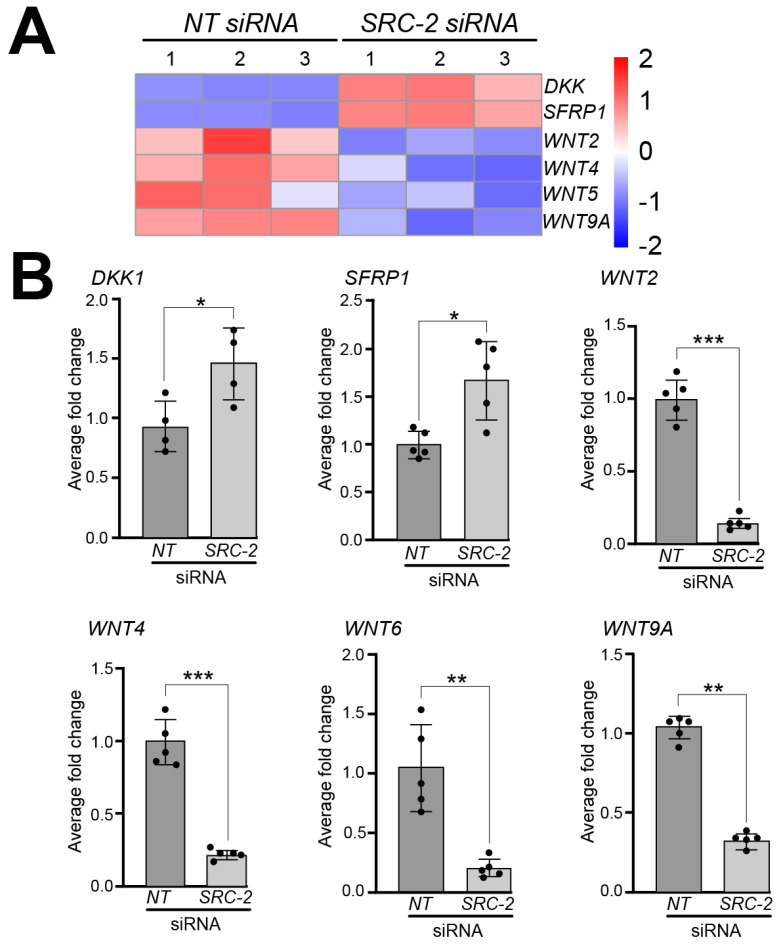
Dysregulated expression of WNT signaling factors in HTR-8/SVneo cells following *SRC-2* knockdown: (**A**) Heat map displays marked changes in the expression of genes associated with WNT signaling between the *NT* siRNA and *SRC-2* siRNA replicate groups. While WNT signaling inhibitors, Dickkopf WNT signaling pathway inhibitor 1 (*DKK1*) and secreted frizzled related protein 1 (*SFRP1*), are upregulated in the *SRC-2* siRNA-treated group, the expression levels of Wnt family member 2 (*WNT2*), *WNT4*, *WNT6*, *WNT8B*, and *WNT9A* are significantly reduced. (**B**) Confirmation by qRT-PCR of the above-described gene expression changes between the *NT* siRNA and *SRC-2* siRNA-treated groups is shown. An explanation of the average fold change (FC ± standard deviation) calculation is described in the Section 2. Results are representative of three independent experiments; * *p*-value < 0.05; ** *p*-value < 0.01, and *** *p* value < 0.001.

A significant number of studies implicate different components of the wingless-type MMTV integration site (WNT) family member signaling pathway in the regulation of human placental development and trophoblast differentiation [71,72]. In our RNA-seq analyses, we found that the expression of a number of WNT signaling members is dysregulated in HTR-8/SVneo cells following *SRC-2* knockdown. For example, our analysis revealed significant decreased expression for the following WNT genes: WNT2, WNT4, WNT6, WNT8B, and WNT9A, and increased expression of the WNT inhibitors: Dickkopf WNT signaling pathway inhibitor 1 (*DKK1*) and secreted frizzled related protein 1 (*SFRP1* (Figure 6A)). At the RNA level, the expression of these genes was validated by qRT-PCR (Figure 6B).

### 3.6. Human Term-Placental Tissue and HTR-8/SVneo Cells Express WNT9A

**Figure 7:**
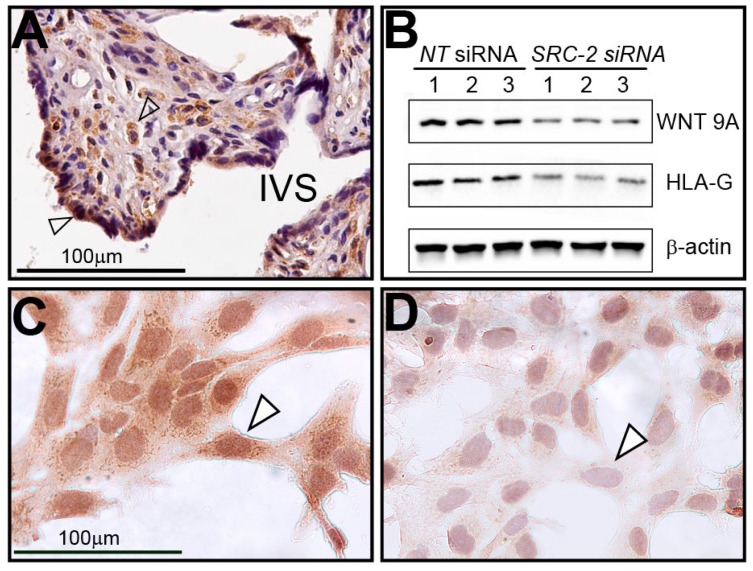
Human term-placental tissue and HTR-8/SVneo cells express WNT9A: (**A**) Immunohistochemistry shows WNT9A is expressed in syncytiotrophoblast cells (white triangle) and stromal cells (open arrow) of chorionic villi; IVS indicates intervillous space. (**B**) Western analysis shows that WNT9A is expressed in HTR-8/SVneo cells previously transfected with *NT* siRNAs. However, WNT9A expression is significantly reduced following *SRC-2* siRNA-mediated knockdown. HLA-G, an established marker in extravillous trophoblast cells and HTR-8/SVneo cells, is also markedly reduced following *SRC-2* knockdown. (**C**) Similar to SRC-2 (see Figure 2), immunocytochemistry shows WNT9A is expressed in the HTR-8/SVneo cells (white triangle). (**D**) Following *SRC-2* knockdown, WNT9A expression is significantly attenuated following SRC-2 knockdown (white triangle). Scale bar in panel (**C**) applies to panel (**D**).

Immunohistochemical analysis reveals that WNT9A is expressed in human chorionic villi in term placental tissue and in cell types that are similar to cells (i.e., syncytiotrophoblasts and stromal cells) that express SRC-2 (Figure 7A). Both Western immunoblotting and immunocytochemistry demonstrate that WNT9A is not only expressed in HTR-8/SVneo cells but that its full expression is dependent on SRC-2 (Figure 7B–D).

### 3.7. Attenuation of WNT9A Expression Significantly Reduces the Sustained Viability, Motility, and Invasiveness of HTR-8/SVneo Trophoblast Cells

**Figure 8:**
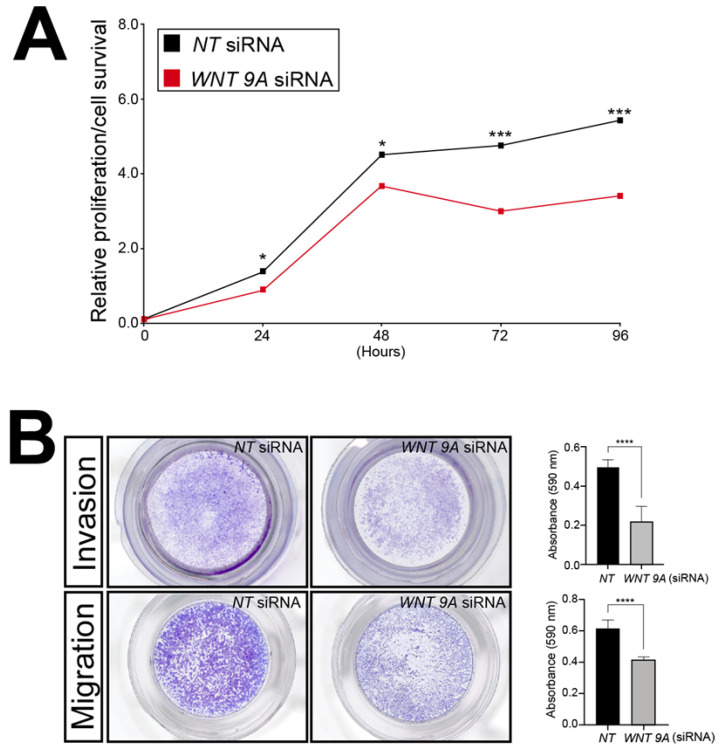
Sustained viability/proliferation, migration, and invasion of the HTR-8/SVneo cell line is compromised following *WNT9A* knockdown: (**A**) The MTT-based assay shows that the number of HTR-8/SVneo viable cells in culture is markedly reduced following *WNT9A* knockdown. (**B**) Top panel row: The transwell invasion assay shows that *WNT 9A* siRNA knockdown results in a significant reduction in the invasive capacity of HTR-8/SVneo cells. Bottom panel row: The transwell migration assay reveals a marked reduction in the migratory capabilities HTR-8/SVneo cells following *WNT 9A* siRNA knockdown. Results in histograms are indicated as the mean ± standard deviation and are representative of three independent experiments; * *p*-value<0.05; *** *p*-value < 0.001; **** *p*-value < 0.0001.

To showcase the utility of this RNA-seq dataset, WNT9A was chosen further for analyses because this Wnt ligand has not been previously associated with trophoblast biology. Accordingly, the functional importance of WNT9A expression to the viability, invasion, and migration properties of the HTR-8/SVneo cells following *WNT9A* siRNA-mediated knockdown was assessed by in vitro assays described above. Similar to SRC-2 (Figure 3), these assays revealed that WNT9A expression is essential for optimum HTR-8/SVneo cellular viability, invasion, and migration (Figure 8).

Collectively, these assays provide functional support for the proposal that WNT9A expression, which is dependent on SRC-2, is essential for trophoblastic functions linked with the development and expansion of the embryonic placenta.

## 4. Discussion

From endometrial receptivity to decidualization, uterine-derived SRC-2 is essential for pregnancy establishment [23]. Studies here are the first to support a possible role for embryonic-derived SRC-2 in pregnancy initiation, suggesting distinct roles for SRC-2 in the maternal and fetal compartments of the placenta during the peri-implantation period. Further underscoring SRC-2’s possible multifunctionality in placental biology, SRC-2 is expressed in many placental cell types—syncytiotrophoblasts, cytotrophoblasts, perivascular fibroblasts, and stromal cells—of the chorionic villus, each known to exhibit specialized roles in ensuring normal placental development and/or function [6].

Using the immortalized first-trimester trophoblast HTR-8/SVneo cell line, established in vitro assays demonstrated an indispensable role for SRC-2 in sustaining HTR-8/SVneo cell viability and in maintaining this cell type’s optimum migratory and invasive properties. From a physiological perspective, these findings are significant as extravillous trophoblasts migrate from the anchoring chorionic villus to invade the maternal decidual basalis to ensure a strong uteroplacental interface [9]. Apart from remodeling the endometrial stromal environment, extravillous trophoblasts transform into endovascular trophoblasts, which convert maternal spiral arterioles into large dilated uteroplacental arteries, essential for normal functioning of the fetomaternal nutrient and gas exchange interface. Impairments in this vasodilation enhancement are attributable to obstetrical complications, such as preeclampsia, fetal growth restriction, and preterm birth [1,16,19,20,73,74,75].

In support of SRC-2 as a potent transcriptional coregulator in the HTR-8/SVneo cell, transcriptomic analyses revealed that the expression levels of a significant number of genes, previously ascribed to the placental phenotype in general or to the trophoblast phenotype specifically, are markedly changed as a consequence of SRC-2 depletion. Interestingly, the expression levels of select members of the WNT signaling pathway are significantly altered when SRC-2 levels are reduced. As a result of this SRC-2 deficit, the net transcriptional changes in WNT signaling components in the HTR-8/SVneo cell result in a reduction in WNT signaling activity. In the context of placental development, canonical WNT-activated signal transduction is known to be pivotal for the renewal of the trophoblast stem cell and its differentiation into syncytiotrophoblasts and extravillous trophoblast cell lineages [72,76,77,78,79]. These studies underscore the proposal that tight coordination between the maintenance of trophoblast stemness and trophoblast stem cell differentiation is critical to the development of a healthy placenta.

Apart from maintaining trophoblastic stemness or specifying a program of trophoblast differentiation, WNT signaling is essential for the functioning—migration and invasion—of the extravillous trophoblast once formed [80,81,82,83,84]. Along with established WNT factors in trophoblast function (WNT2, WNT4, WNT6, WNT8B, DKK1, SFRP1 [81,85,86,87,88,89,90,91], we reveal that the expression of WNT 9A is reduced with SRC-2 knockdown. A paralog of WNT 9B, WNT9A (initially named WNT 14), is a secreted WNT ligand driving both canonical and non-canonical WNT signaling pathways [92]. Zebrafish and mouse model studies have shown that WNT 9A activity is linked to a broad spectrum of physiologies, ranging from specification of hematopoietic stem cells from embryonic stem cells, embryonic patterning, to organogenesis [92]. Although there is a limited understanding of WNT 9A in the trophoblast cell lineage, early studies document WNT9A expression in the trophectoderm of the murine embryo [93]. Interestingly, analysis of recent transcriptomic datasets derived from placental tissue biopsied from pregnancies of healthy normotensive women (n = 147) and women diagnosed with preeclampsia (n = 91) [94] show that WNT 9A along with SRC-2 are significantly reduced in placenta tissue from preeclampsia patients (WNT 9A: (Log2FC(DESeq2) = −0.22; adjusted *p*-value 0.032); SRC-2: (Log2FC(DESeq2) = −0.09; adjusted *p*-value: 0.029) https://www.obgyn.cam.ac.uk/placentome [94]; accessed 4 October 2024). Given that the activities of SRC family members and WNT signaling intersect in other physiological systems [95,96,97] along with our cell-based results here, it is not unreasonable to propose that WNT 9A may represent one of a number of WNT activation transduction pathways through which SRC-2 controls extravillous trophoblast cell migration and invasion.

Using the HTR-8/SVneo cell model, previous studies have shown that SRC-3, a close relative of SRC-2, is critical for HTR-8/SVneo cell invasive and migratory functions, whereas, unlike SRC-2 (Figure 2A), SRC-3 is not required for HTR-8/SVneo cell viability [30]. These studies also showed that SRC-3 directly interacts with AKT as a mechanism by which SRC-3 regulates HTR-8/SVneo cell migration and invasion. Interestingly, our transcriptomic studies predict that the PI3K/AKT signaling pathway may also be adversely affected with SRC-2 depletion in the HTR-8/SVneo cell. Future studies will address this proposal along with addressing whether SRC-2 is critical for the development of the syncytiotrophoblast and/or extravillous trophoblast cell lineages from the cytotrophoblastic stem cell through use of the human trophoblast stem cell model [98,99].

## 5. Conclusions

Our investigations highlight an unsuspected role for SRC-2 in trophoblastic cell functions that are known to drive mammalian placentation. Cell-based assays paired with transcriptomic analysis reveal that select components of the WNT signaling pathway can mediate SRC-2’s role in maintaining the sustained viability, motility, and invasion of the extravillous trophoblast. Together, these findings support an indispensable role for SRC-2 and its WNT effectors in maintaining optimum functioning of the extravillous trophoblast, a placental cell type that is essential for early pregnancy establishment.

## Figures and Tables

**Table 1:** List of human TaqMan expression assays used in these studies.

*Gene Name*	*Gene ID*	*Catalog Number*
*ALDH1A1*	*216*	*Hs00946916_m1*
*CAPN6*	*827*	*Hs00560073_m1*
*CCR7*	*1236*	*Hs01013469_m1*
*CDKN1C*	*1028*	*Hs00175938_m1*
*DIO2*	*1734*	*Hs05050546_s1*
*DKK1*	*22943*	*Hs00183740_m1*
*FSTL3*	*10272*	*Hs00610505_m1*
*FOXO4*	*4303*	*Hs00172973_m1*
*HLA-G*	*3135*	*Hs00365950_g1*
*LAMB3*	*3914*	*Hs00165078_m1*
*NCOA2*	*10499*	*Hs00896109_m1*
*SFRP1*	*6422*	*Hs00610060_m1*
*WNT2*	*7472*	*Hs00608224_m1*
*WNT4*	*54361*	*Hs01573505_m1*
*WNT6*	*7475*	*Hs00362452_m1*
*WNT 9A*	*7483*	*Hs01573829_m1*
*HPRT*	*ThermoFisher Scientific Inc.*	*4333768T*

## Data Availability

The data from these studies are available following a reasonable request to the corresponding author.

## Supplementary Materials

The following supporting information can be downloaded at: https://www.mdpi.com/article/10.3390/cells14131024/s1, Figure S1. Human pregnancy-term placental tissue co-express hCG and SRC-2. (A) Human chorionic villi stained for DAPI (open triangle). (B) Same section immunofluorescently stained for hCG (green; open triangle). (C) Same section immunofluorescently stained for SRC-2 (red; open triangle). (D) Merged image of images in (B) and (C); note the colocalization of hCG and SRC-2 (open triangle). Scale bar in (A) applies to all panels. Figure S2. (A–C) Dot plots of enriched genes within the DEG dataset between the *NT* siRNA and *SRC-2* siRNA transfected HTR-8/SVneo groups are categorized according to biological processes, cellular components, and molecular functions respectively. (D) Analysis by KEGG reveals top enrichment for proteins involved in PI3K-AKT signaling, cell adhesion, protein digestion and absorption, and extracellular matrix-receptor interactions. Figure S3. (A) Heat map showing the difference in expression of *ALDH1A1*, *CCR7*, and *LAMB3* in HTR-8/SVneo cells following *NT* siRNA or *SRC-2* siRNA knockdown. (B) Histograms show the quantitative differences in expression of *ALDH1A1*, *CCR7*, and *LAMB3* between the *NT* siRNA and *SRC-2* siRNA treated HTR-8/SVneo groups. Results in histograms are indicated as the mean ± standard deviation and are representative of three independent experiments; ** *p*-value < 0.01; *** *p*-value < 0.001; **** *p*-value < 0.0001. Table S1. Differentially expressed genes between the NT siRNA and SRC-2 siRNA-transfected groups.
