# Supplementary material for: Human Extravillous Trophoblasts Require SRC-2 for Sustained Viability, Migration, and Invasion
Source: Cells. 2025 Jul 4;14(13):1024. doi: 10.3390/cells14131024 (PMC12249151; doi:10.3390/cells14131024)
Supplement: Supplementary file 1 [file cells-14-01024-s001.zip › cells-3645076-supplementary.pdf]

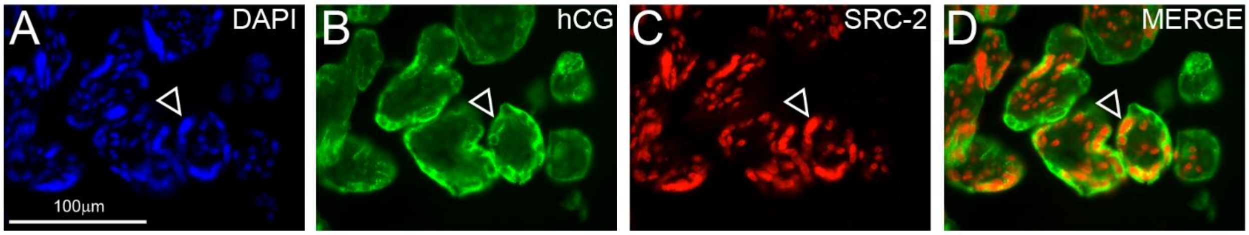

Figure S1. Human pregnancy-term placental tissue co-express hCG and SRC-2. (**A**) Human chorionic villi stained for DAPI (open triangle). (**B**) Same section immunofluorescently stained for hCG (green; open triangle). (C) Same section immunofluorescently stained for SRC-2 (red; open triangle). (D) Merged image of images in (**B**) and (**C**); note the colocalization of hCG and SRC-2 (open triangle). Scale bar in (A) applies to all panels.

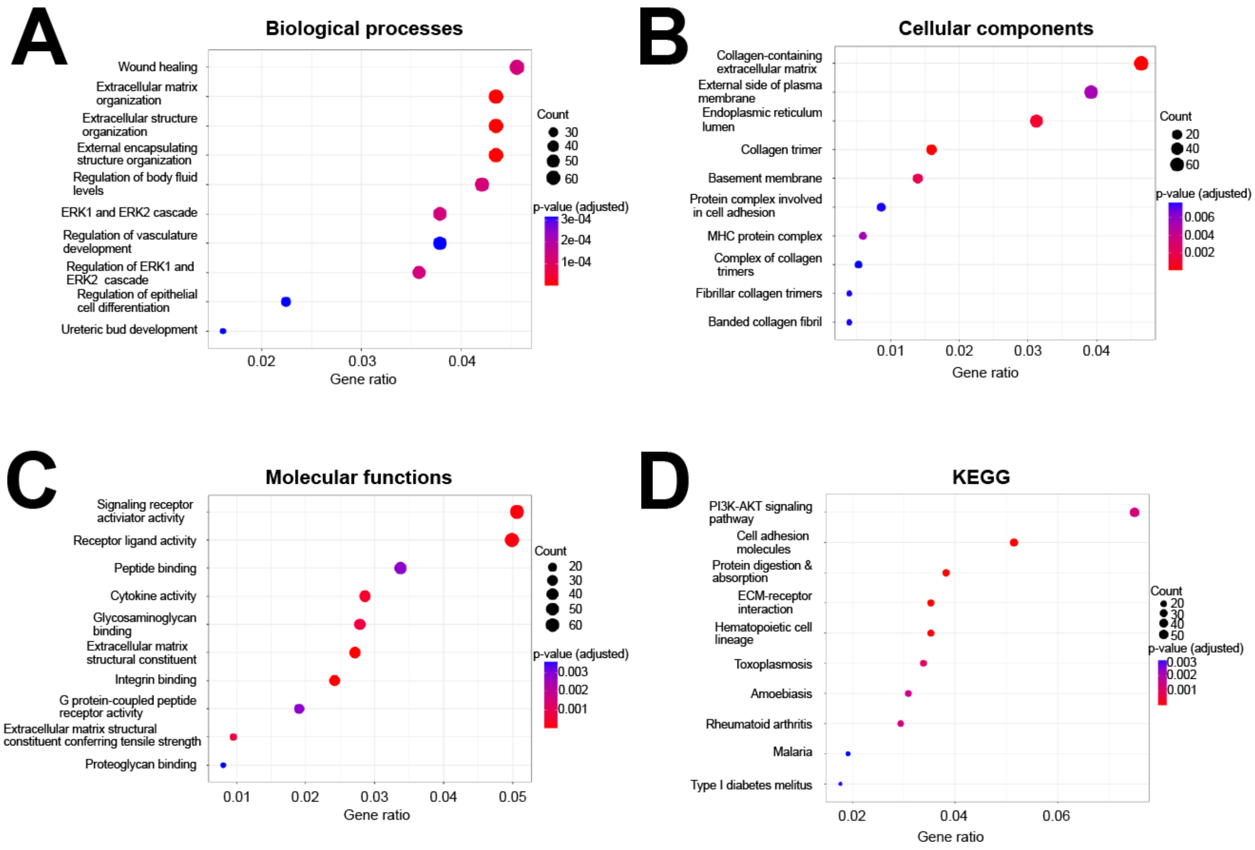

Figure S2. (**A-C**) Dot plots of enriched genes within the DEG dataset between the *NT* siRNA and *SRC-2* siRNA transfected HTR-8/SVneo groups are categorized according to biological processes, cellular components, and molecular functions respectively. (**D**) Analysis by KEGG reveals top enrichment for

proteins involved in PI3K-AKT signaling, cell adhesion, protein digestion and absorption, and extracellular matrix-receptor interactions.

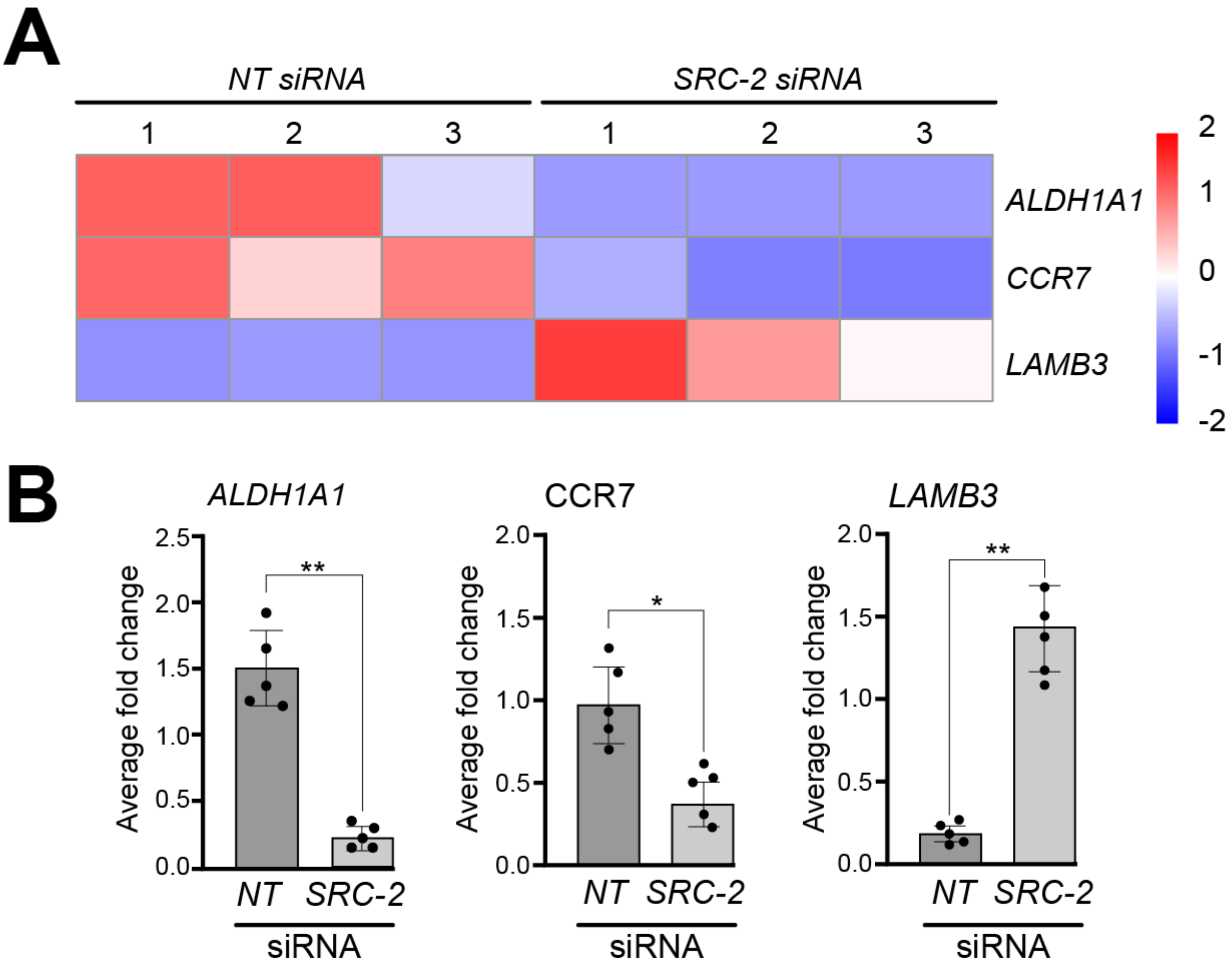

Figure S3. (**A**) Heat map showing the difference in expression of *ALDH1A1*, *CCR7*, and *LAMB3* in HTR-8/SVneo cells following *NT* siRNA or *SRC-2* siRNA knockdown. (**B**) Histograms show the quantitative differences in expression of *ALDH1A1*, *CCR7*, and *LAMB3* between the *NT* siRNA and *SRC-2* siRNA treated HTR-8/SVneo groups. Results in histograms are indicated as the mean ± standard deviation and are representative of three independent experiments; ***p*-value<0.01; ****p*-value <0.001; *****p*-value<0.0001.
